# Diseases of the Fingers D.—Diseases of the Phalangeal Bones and Joints

**Published:** 1891-08-29

**Authors:** 


### SURGERY.

### DISEASES OF THE FINGERS (*Continued*).

D.—Diseases of the Phalangeal Bones and Joints.

*Strumous dactylitis* is a common affection amongst badly-fed and unhealthy children. There is sometimes a history of a blow or strain, but not always. Probably, however, this is generally the beginning of the disease, although the feeble health is the predisposing cause. It is usually described as a "rarefying caries" running a chronic course, but this is not quite correct according to Mr. Walson Cheyne, who considers the condition one of osteomyelitis, or inflammation beginning in the soft parts of the bone. The part affected slowly enlarges; the finger becomes stiff, and the skin dusky red and œdematous. There is pain at first, but this soon lessens, and when the disease is advanced it is usually painless. Sinuses ultimately form, and unless the part is treated the finger will be lost. As these cases do not come under treatment usually until the advanced stage, it is generally necessary to amputate, but scraping out the diseased bone from the centre and leaving the periosteum is sometimes successful.

*Syphilis* attacks the finger-bones in two ways, (1) as a *gumma* or deposit, and (2) as an infiltration of the parts near the joints. In the first case it is generally the proximal bone that is diseased. The caries which is set up invades the nearest joint, and the result is usually ankylosis. It is most common in babies affected with the hereditary taint, and attacks those who are particularly weak and puny. Treatment has to be directed to the general health, and mild mercurials (such as in unction with unguent hydrarg.) should be supplemented by tonics if the child is old enough, and by careful feeding if very young.

The second variety of syphilitic disease occurs as a smooth swelling, near one of the joints, either in early childhood or (more rarely) as a result of the acquired disease. The tissue affected appears to be the fibrous part of the ligaments, synovial membrane and periosteum. The result is a stiff joint in most cases. Both the above two affections are somewhat rare.

*Enchondroma.*—Cartilaginous growths of the fingers are fairly common in young people about the age of puberty. They are usually multiple and grow from the shafts of the phalanges, not from the articular ends, but nearer the extremities than the centre of the shaft. Cohnheim considers that they start from the remains of the hyaline temporary cartilage, but this requires proof. They are almost always distinctly vascular, and contain myxomatous tissue more often than is generally supposed, probably. Their appearance is blue or purple, and their consistence soft and spongy. It is easy to remove them by an incision and a little scraping, but they are apt to recur. They do not do so, however, indefinitely, as the tendency to these tumours seems to cease when adult life is reached.

*Osteoma.*—The firmer varieties of osseous out-growths rarely occur on the fingers, but small nodules sometimes appear under the nail after inflammatory affections in the neighbourhood (matrixitis, &c.) The periosteum probably becomes vascular and active, and the small formation is the result.

The *spongy* or *cancellous* osteomata occasionally grow from the epiphyseal cartilages at the base of the phalanges. They are usually multiple, and the tendency to them is inherited. They naturally occur in early life, when the cartilage is active, and when it often contains small secondary centres, as in the epiphyses of the larger bones in rickets. It is probable that the so-called enchondromata of the fingers are in reality often osteomata. It should be remembered that the latter almost always start from the cartilage, the former from the shaft of the bone.

*Sprains* of the fingers require very careful examination, for it is not uncommon to get (1) Separation of the epiphysis, (2) Rupture of a tendon (especially the extensor), or (3) partial dislocation of the "glenoid" plate. These are not easily recognised in all cases, hence it is important to get the injured part into good position on a splint, and allay inflammation by cooling lotions, &c.

Rupture of the extensor tendon occurs usually (when from over-strain) close to its insertion, and the deformity due to unrestrained action of the flexors does not immediately appear in many cases. Operative interference is not generally of any avail; the splint is the best treatment. The reason of this is that the insertion of the tendon is flattened and non-vascular, and the tear is irregular and only partial; hence the difficulty of suturing with success.

*Separation of the Epiphysis*, as stated in an earlier paper, gives rise to little, if any, deformity in many instances, owing to the ligaments and tendons acting as splints. Bad results, however, seldom follow. The glenoid plate in front of the inter-phalangeal joints may be injured by sudden wrenches, and a stiff finger may result. These untoward accidents cannot be easily diagnosed; but if a well-padded wooden splint be carefully applied to the finger and rest enforced, the surgeon has put the part into the best condition for recovery.

*Fracture* of the phalanges must be treated like other broken bones; but it is obvious that early passive movement must be employed to avoid stiffness, the inflammatory state preceding and accompanying callus-formation in such small bones readily extending to the joints and causing fibrous ankylosis. Fractures of the metacarpal bones are sometimes difficult to reduce owing to the thickness of the surrounding tissues and the difficulty of straightening. As they leave behind great impairment of the hand if the fragments are allowed to remain in position, it is advisable, if there is any difficulty, to give an anæsthetic.

*Compound Dislocations* frequently occur, the proximal end of the phalanx projecting through the wound. The prognosis in these cases is unfavourable. However carefully the parts are cleansed and reduced, it is only too common for violent cellulitis and necrosis to follow, necessitating amputation. In such cases the parts should not only be washed with an antiseptic, but, if possible, some of the solution should be syringed into the wound, which should not be stitched up.

